# Levofloxacin and Rifampin Resistance in *Helicobacter pylori* Isolates from Central-Western Colombia: Role of *gyrA* Mutations in Fluoroquinolone Resistance

**DOI:** 10.3390/antibiotics15050452

**Published:** 2026-04-30

**Authors:** Adalucy Álvarez-Aldana, Leonardo Beltrán-Angarita, Yina Marcela Guaca-González, Manuel Alejandro Velandia-López, Lyudmila Boyanova

**Affiliations:** 1 Grupo de Investigación en Microbiología y Biotecnología (MICROBIOTEC), Programa de Microbiología, Facultad de Ciencias de la Salud, Exactas y Naturales, Universidad Libre, Pereira 660001, Colombia; manuel.velandial@unilibre.edu.co; 2 Grupo de Investigación GRIEPI, Facultad Ciencias de la Salud, Unidad Central del Valle del Cauca, Tuluá 763021, Colombia; lbeltran@uceva.edu.co; 3 Grupo de Investigación Infección e Inmunidad, Programa de Medicina, Facultad Ciencias de la Salud, Universidad Tecnológica de Pereira, Pereira 660001, Colombia; 4 Grupo Investigación Enfermedades Infecciosas (GRIENI), Programa de Medicina, Facultad Ciencias de la Salud, Universidad Tecnológica de Pereira, Pereira 660001, Colombia; yimagugo@utp.edu.co; 5 Department of Medical Microbiology, Medical University of Sofia, 2 Zdrave St., 1431 Sofia, Bulgaria

**Keywords:** *Helicobacter pylori*, antibiotic resistance, *gyrA* mutations, levofloxacin, rifampin, Colombia

## Abstract

**Background:** *Helicobacter pylori* causes one of the most prevalent chronic bacterial infections worldwide. Data about *H. pylori* resistance rates to levofloxacin and rifampin (antimicrobials for second-, third- or fourth-line therapy) in Colombia and South America are scarce. The present study aimed to assess levofloxacin and rifampin resistance rates among 61 *H. pylori* isolates from the western-central region of Colombia and to identify *gyrA* point mutations associated with levofloxacin resistance. **Methods:** For this purpose, antimicrobial susceptibility was determined using the E-test method and *gyrA* mutations in levofloxacin-resistant isolates were identified by PCR amplification followed by DNA sequencing. **Results:** Resistance rates to levofloxacin and rifampin were 24.6% and 13.1%, respectively. Among the 15 levofloxacin-resistant isolates, 6 (40%) isolates had ≥2 *gyrA* mutations and 5 of them exhibited high (≥32 mg/L) levofloxacin MICs. In addition, two new mutations (Y90C and V89D) were also detected. **Conclusions:** Clinically relevant resistance to levofloxacin and rifampin was detected among *H. pylori* isolates from central-western Colombia. Multiple *gyrA* mutations were identified in a significant proportion of levofloxacin-resistant isolates and were mainly associated with high MIC values, highlighting the need for regional surveillance to guide eradication therapies.

## 1. Introduction

*Helicobacter pylori* is the second most common causative agent of bacterial infections in humans [1]. *H. pylori* is the most frequent cause of chronic gastritis and may lead to severe gastroduodenal pathologies in some patients, including gastric and duodenal peptic ulcer disease (PUD), gastric cancer, and gastric mucosa-associated lymphoid tissue (MALT) lymphoma [2], and over half of the people worldwide, including approximately 70% of those in Africa and South America, have been infected [1].

Eradication of *H. pylori* is a key strategy to reduce the risk of the associated diseases [3]. In the algorithm for empirical eradication of *H. pylori,* if individual susceptibility testing results are not available, and if resistance to clarithromycin is less than 15%, levofloxacin can be used as a second-line antibiotic and rifabutin as a fourth-line antibiotic. In the case of clarithromycin resistance above 15%, levofloxacin is a second-line antibiotic and rifabutin is either a third- or fourth-line antibiotic [4]. Resistance to the most effective antibiotics used in *H. pylori* eradication regimens is increasing globally, leading to significant failure rates of current eradication therapies. There are considerable variations between countries. Resistance to levofloxacin ranged from 3.3 to 65.7%, and that to rifampicin, with relatively scanty reports of resistance determination, ranging from 4.4 to 90% [5].

Evaluating *H. pylori* resistance in the period 2008–2023, Cabrera et al. [6] found a wide range of resistance rates even in Latin American children and adolescents for clarithromycin (8.1 to 79.6%), metronidazole (1.9 to 40.2%), and amoxicillin (0% to 10.4%), and levofloxacin resistance for about 3%.

Genotypic assessment of antimicrobial resistance has revealed molecular mechanisms of *H. pylori* resistance to antimicrobials. The mechanism of fluoroquinolone resistance in *H. pylori* has been identified to be linked to mutations in the quinolone resistance-determining regions (QRDR) of the *gyrA* and *gyrB* genes, coding for the DNA gyrase. Single- or dual-point mutations in *gyrA* had a greater impact, while *gyrB* frequently occurred alongside *gyrA* mutations [7,8]. In the study of Zerbetto De Palma et al. [8] in Argentina, amino acid substitutions in the *gyrA* gene at positions 91 (D91G, N, A, Y or H) and 87 (N87L, I, A or K) were the most common substitutions linked to levofloxacin resistance. However, substitutions at other positions, such as 88 (A88V or P) or 86 (D86N), were also detected [8].

Given the increasing prevalence of antimicrobial resistance, tailored treatment based on genotypic detection of *H. pylori* resistance of the infected patient’s strains to the antimicrobial agents appears to be a reasonable alternative. This study aimed to determine levofloxacin and rifampin resistance rates of *H. pylori* isolates from a collection of the western-central Colombian region and assess *gyrA* mutations in levofloxacin-resistant isolates.

Levofloxacin and rifampin were chosen because, in the era of increasing antibiotic resistance, they have an important role as agents in either the second-line or rescue therapy of *H. pylori* infection, and resistance to them can lead to eradication failure.

## 2. Results

### 2.1. Isolates

This collection was obtained from a previous study in which 115 patients who met the inclusion criteria were recruited, of whom 61 patients were culture-positive for *H. pylori* (53.0%). The isolates were from 21 males and 40 females with a mean age of 41.8 years, ranging from 18 to 70 years. A total of 8 of the 61 patients reported having received prior treatment between 9 and 15 months before this study; however, most of them (7 out of 8) were unable to describe the complete eradication therapy they had received, as mentioned in a previous study [9]. All these isolates (100%) were recovered and used in the present study.

### 2.2. Phenotypic Resistance by the E-Test Method

MICs for 61 isolates were determined by E-test, showing that 15 and 8 isolates were resistant to levofloxacin and rifampin, respectively. Overall, 24.6% of all isolates exhibited MICs of >1 mg/L of levofloxacin, 60% (9/15 isolates) of which had MIC ≥ 32 mg/L, and 13.1% had rifampin MICs of >1 mg/L, of which 75% (6/8) had MIC ≥ 32 mg/L. Table 1 presents an overview of the resistance rates, the number of resistant isolates and the distribution of MICs (mg/L) in the 61 isolates. Dual resistance was found in 4.9% (3/61 isolates). A total of 8 of 61 isolates were detected from patients who had received prior treatment for *H. pylori* infection. Of this group, seven isolates showed secondary resistance to levofloxacin (37.5%) with MICs ranging between 3 and 32 mg/L.

### 2.3. Genotypic Resistance

The *gyrA* gene of the 15 levofloxacin-resistant isolates was amplified and substitutions were found in positions 87, 89, 90, and 91 of the sequences (Table 2). The mutations detected were N87I, V89A/D, Y90A/C and D91G/M/N. In 3 of the 15 levofloxacin-resistant isolates, showing levofloxacin MICs of 2, 3 and 32 μg/mL, no mutation was detected.

In 6 of the 15 levofloxacin-resistant isolates, a change in one amino acid (N87I, V89D, Y90C, D91G, and D91N) was found. Mutations at positions 91 and 89 were related to low MICs ≤ 8 mg/L, while those at positions 87 and 90 were associated with higher MICs of ≥32 mg/L. In 6.7% (1/15) of the isolates, a double mutation (V89A and Y90A) was observed, and in 33.3% (5/15), there were triple mutations (V89A, Y90A and D91M). Against five of the six isolates with double or triple mutations, levofloxacin MICs were ≥32 mg/L.

## 3. Discussion

*H. pylori* infection is frequently acquired during childhood, becoming lifelong if not successfully eradicated [10]. Over the last few years, the eradication has become more complicated due to the increasing antibiotic resistance rates in *H. pylori* worldwide [11]. Traditionally, different combined regimens have been used to eradicate the infection, including a proton pump inhibitor (PPI) or (more recently) vonoprazan to increase gastric pH, plus two or three antibacterial agents, sometimes with the addition of bismuth preparations. Amoxicillin, metronidazole, clarithromycin, tetracycline and levofloxacin are the most frequent antibiotics used in different combinations in eradication regimens [12].

Due to the increasing antibiotic resistance, other compounds, such as the bactericidal antibiotics rifabutin and rifaximin in the rifamycin class, have been used for treatment [13]. This antibiotic class has traditionally been used for the treatment of tuberculosis [14]. According to the Maastrich VI algorithm for empirical *H. pylori* eradication, these antibiotics have been considered for third- or fourth-line eradication therapy [4].

Rates of rifampin resistance varied in different countries and studies. A recent analysis carried out in Chinese minorities revealed a high rate of resistance to rifampin, up to 57.61%, in 276 individuals studied [15]. This is similar to another study in the Tibetan Autonomous Region of China, in which a resistance rate of 73.2% was found in 153 isolates [16]. In Egypt, among 134 samples from dyspeptic adults, a high resistance rate (up to 90%) to rifampin was found [5,17]. On the other hand, rifampin resistance reports have been infrequent in European countries. For example, the associated resistance rates were 2.2% in Germany [18], 0.9% in France [19], and 2.8% in Switzerland [20]. In a review article on *H. pylori* antibiotic resistance in Europe (2018), the rifampin resistance rate was only around 0.9% [21]. Likewise, in Israel, isolates from patients with Arab and Jewish ancestry were tested for rifampin resistance and exhibited resistance rates of 4% and 1.2%, respectively [22]. In Latin America, these studies were scarce; one analysis performed in Ecuador evaluated *H. pylori* resistance rates to different antibiotics. However, the authors did not find isolates resistant to rifabutin [23], similarly to two studies in Colombia, in which the isolates did not show rifampin resistance [24,25].

In the present study, the rifampin resistance rate (13.1%) was higher than that (often <5–7%) usually reported in Europe [18,19,20,21]. However, the rifampin resistance has been determined *in vitro* only to detect that for rifabutin, which can be used for *H. pylori* eradication. The rifabutin resistance has generally been lower (0% to <4%) than that of rifampin [19,20,26]. When comparing the results obtained in a previous study performed on isolates from the period between February and October 2018 [27] with the current results, a 1.8-fold increase in rifampicin resistance was observed in just three years, rising from 13.1% in the current study (isolates from 2015) to 23.8% in the previous study (isolates from 2018) [27]. For this reason, it is important to conduct surveillance in the region in order to analyze the presence and trend of the resistance to rifampin.

Levofloxacin-based regimens have been used as second- and third-line eradication therapy [3,4,5]. Fluoroquinolones have also been used as first-line regimens to replace clarithromycin in macrolide-based triple therapy. Branquinho et al. [28] evaluated a levofloxacin-based sequential regimen as a first-line therapy and reported a suboptimal cure rate of 79%. Ciccaglione et al. [29] used a triple regimen of esomeprazole + amoxicillin + levofloxacin without or with the addition of bovine lactoferrin as a first-line therapy for *H. pylori* eradication. The authors obtained high eradication success (96.1%) with the regimen with lactoferrin addition [29].

Fluoroquinolone resistance can significantly affect the success of fluoroquinolone-based regimens. In the study of Nishizawa et al. [30], *H. pylori* cure rate dropped by >66% (from 100.0% to 33.3%) in isolates with gatifloxacin resistance and *gyrA* mutations when compared to those susceptible to gatifloxacin and lacking *gyrA* mutations.

Levofloxacin resistance rates in *H. pylori* have widely varied from about 3.3% to >78% [5,15,16,17,18,31]. Data about the prevalence of levofloxacin resistance in Colombia have been described in different works. Reports in Colombia displayed a resistance rate of 27.3% in 2014 (Bogotá) [32] and 20.3% in 2021 (Tuquerres and Tumaco) [24]. The results in the present analysis were similar to previous reports in the country, but differed from a study with patient isolates in the city of Bogotá (resistance rate of 48.6%) [33]. In our previous study [27], we reported for the first time *H. pylori* multidrug resistance in 14.3% in the western-central region of Colombia.

Our results on *H. pylori* resistance to levofloxacin were comparable to those reported in Bulgaria (overall resistance rate, 19.4%), and slightly lower than those of primary resistance in China (28%) [34,35]. However, the levofloxacin resistance rates (24.6%) in our work were higher than those observed in the Americas region (primary resistance in 15%, and secondary resistance in 22%), reported in a meta-analysis of the regions of the World Health Organization [36]. When comparing the results obtained in a previous study [27] with those in the current one, a relative increase of 6% in levofloxacin resistance was observed, rising from 24.6% in the current study (isolates from 2015) to 26.2% in the previous study (isolates from 2018). The lack of detectable mutations in three levofloxacin-resistant isolates may be due to either different or newer mutations.

We had complete agreement between the results of the phenotyping and genotyping testing of the isolates. The mechanism of fluoroquinolone resistance in *H. pylori* is related to mutations in the QRDR (the quinolone-resistance-determining region) of the *gyrA* and *gyrB* genes, which encode the DNA gyrase. Single-point or dual mutations in *gyrA* had the greatest impact, whereas in *gyrB,* they frequently occurred simultaneously with the *gyrA* mutation. The reported *gyrB* gene mutations are rare or do not appear to play a major role in fluoroquinolone resistance in *H. pylori* [7,8,23,24,32,33]. Variants associated with levofloxacin resistance are more commonly found in the *gyrA* gene, while variants in the *gyrB* gene are less frequently associated with resistant strains; for example, in the study of Camorlinga-Ponce et al. [37], variants in the *gyrB* gene were observed in two resistant strains but were also present in four of the sensitive strains [37].

Mutations outside the QRDR rarely cause fluoroquinolone resistance [38]. However, efflux pumps, such as HefDEF (HP0971-HP0969) and HefGHI, can confer resistance to different antibiotics, including levofloxacin [39].

In the present study, 6 of 15 levofloxacin-resistant isolates had single mutations such as N87I, D91N and D91G, which have been considered the most prevalent mutations [8]. However, when only mutations at position 91 were present, levofloxacin MICs against the three strains were from 1.5 to 4 mg/L, unlike the results of Zerbetto De Palma et al. [8], revealing a wide MIC range. The N87I mutation was observed in only one isolate and was associated with a high MIC (≥32 mg/L), consistent with results from other studies [40].

Among the levofloxacin-resistant isolates, two fifths carried more than one mutation, including triple amino acid mutations (V89A, Y90C, D91M in 33.3% of the isolates) and double mutations (V89D and Y90A, in 6.7%). Moreover, five out of the six isolates with more than one *gyrA* mutation were associated with high levofloxacin MICs (≥32 mg/L), like in the results of López-Gasca et al. [31].

In the publication of Mannion et al. [24] reporting a resistance to levofloxacin of 20.3% (12/59 isolates) in a cohort coming from southeastern Colombia, all resistant samples had a mutation in the gene *gyrA*, at the amino acid 87 or 91 [24]. By observing the situation in other Latin American countries, several studies have found high resistance rates in *H. pylori* to levofloxacin, and all of them pointed to *gyrA* gene mutations being involved in this phenotype. For example, in Venezuela, genetic analyses showed the presence of mutations in the *gyrA* gene at amino acid positions 87 (N87T or N87I) and 91 (D91N), and the change N87I and the double mutation N87T/D91N were related to the levofloxacin resistance [30]. In Ecuador, a genetic study in *H. pylori* isolates showed that the resistance was associated with mutations in the *gyrA* gene (N87K, N87I, N87T, D91N and D91G) [23]. Moreover, in Mexico, 17 out of 22 resistant strains had point mutations at amino acid positions 87 and 91 of the *gyrA* gene [37].

Importantly, in the present study, mutations in the QRDR, which have not previously been reported in the literature, such as Y90C and V89D, were detected and were linked to levofloxacin MICs of ≥32 mg/L and 8 mg/L, respectively. Indeed, the significance of these new mutations needs to be investigated and functionally validated in future studies.

### Limitations of the Study

Given that antibiotic resistance rates may change over time, the resistance patterns in the present study may not fully reflect the current situation in the country. Although the study was conducted years ago and could not be updated for complex reasons, it presents the frequency and influence of multiple mutations that can compromise the success of treatment for *H. pylori* infection when second, third- or fourth-line eradication regimens are used. Despite analyzing isolates from only 61 patients in two Colombian cities, the results highlight the need to perform *in vitro* susceptibility testing for fluoroquinolones and rifampicin before using them for *H. pylori* eradication.

## 4. Materials and Methods

### 4.1. Specimens

For this prospective study, the *H. pylori* isolates were obtained from individuals with gastroduodenal diseases who participated in gastroenterological examination in two cities of the western-central region of Colombia (Pereira and Manizales) during 2015. The study included 61 consecutive patients, of whom 40 were women and 21 were men. Of them, 40 subjects were from Pereira and 21 from the nearby city of Manizales [9]. According to the diagnosis, 58 had chronic gastritis (of which 5 had atrophic gastritis), one subject had a gastric ulcer, one had a duodenal ulcer, and one patient had intestinal metaplasia. We do not have detailed information on whether and with what regimens the patients had been treated for *H. pylori* infection.

Inclusion criteria were (i) no previous use of proton pump inhibitors, anti-H2 inhibitors, or antibiotics for four weeks prior to the study, and (ii) voluntary signature of informed consent. These isolates were previously tested for antibiotic susceptibility (for metronidazole, clarithromycin, amoxicillin, and tetracycline) as described in a previous study [9]. This study was approved by the bioethics committee (CBE-UTP) of the Faculty of Health Sciences of the Universidad Tecnológica de Pereira. This work was performed under the guidelines of resolution no. 8430/1993 of the Health Ministry of Colombia and the Declaration of Helsinki.

The isolates were kept frozen at −80 °C and after being de-frozen, they were plated (100 µL per isolate) onto culture media Tryptic soy agar (TSA, Oxoid, Hampshire, UK or Merck, Whitehouse Station, NJ, USA), supplemented with sheep blood (7%), BD BBL™ IsoVitaleX™ (0.5%) (Becton, Dickinson and Company, Sparks, MD, USA) and were incubated under microaerophilic conditions (Thermo Scientific™ Oxoid™ CampyGen™, Thermo Fisher Scientific, Waltham, MA, USA) at 37 °C for 5 to 7 days. Colonies were confirmed with Gram staining and biochemical tests (positive urease, catalase and oxidase test) [41].

### 4.2. Detection of Phenotypic Resistance by the E-Test Method

The isolates that were confirmed as *H. pylori* were subcultured in TSA with 7% sheep blood and 0.5% Isovitalex. Then, the isolates were susceptibility tested by E-tests (AB BIODISK North American Inc., Piscataway, NJ, USA) for levofloxacin and rifampin. Suspensions from pure 48 h subcultures were prepared in Brucella broth supplemented with 0.5% Isovitalex and inoculum turbidity was adjusted to McFarland 3.0 standard. Thereafter, they were plated onto TSA plates supplemented with sheep blood (7%) and BD BBL™ IsoVitaleX™ (0.5%) (Becton, Dickinson and Company, Sparks, MD, USA). E-test strips were placed and incubated under microaerophilic conditions at 37 °C for 72 h. Antimicrobial susceptibility was detected as minimum inhibitory concentrations (MICs). *H. pylori* strain ATCC 43504™ was used as a control. Levofloxacin and rifampin do not have established CLSI interpretive criteria, so resistance to levofloxacin and rifampin was interpreted based on EUCAST breakpoints (levofloxacin ≥ 1 mg/L, and rifampin ≥ 1 mg/L) [42].

### 4.3. Detection of Genotypic Resistance

Genomic DNAs were obtained from pure cultures with commercial kits according to the provider’s instructions (QIAamp^®^ DNA Mini and Blood Mini Handbook, Qiagen, Hilden, Germany). Sequencing was performed only on the isolates phenotypically resistant to levofloxacin by the E-test. Sanger sequencing was performed to detect mutations related to levofloxacin resistance. A 569 bp fragment of the *gyrA* gene was assessed with the primers 32fw (5′-ATA TTG TAG AAG TGG GGA TTG AT-3′) and 600rv (5′-ATG CAC TAA AGC GTC TAT GAT T-3′) [43]. Briefly, for Sanger sequencing, the PCR products were first purified with ExoSAP (Thermo Fisher Scientific, Waltham, MA, USA), and the samples were then reamplified using a single primer for each antibiotic to be evaluated. A second purification with SAM Solution and X-Terminator Solution (Thermofisher, Waltham, MA, USA) was carried out. The amplification products were injected onto the DNA ABI Prism 3100 Avant Genetic Analyzer (Thermo Fisher Scientific, Waltham, MA, USA) and the results were analyzed using software Sequencing Analysis 5.1.

### 4.4. Statistical Analysis

For all analyses, the VassarStats webpage (http://vassarstats.net/) was used for establishing resistance frequencies and 95% confidence intervals.

## 5. Conclusions

Clinically significant resistance to either levofloxacin or rifampin was observed in *H. pylori* isolates from central-western Colombia, impacting over 24% and approximately 13% of the isolates, respectively. Additionally, two novel mutations, Y90C and V89D, were identified and linked to elevated levofloxacin MICs, ranging from 8 to ≥32 mg/L. Further investigation is required to determine the clinical importance of these mutations. A substantial number of levofloxacin-resistant isolates exhibited multiple *gyrA* mutations, frequently correlating with high MIC values. The current study’s findings demonstrate the presence and impact of several mutations that can jeopardize the effectiveness of *H. pylori* treatment when second-, third-, or fourth-line eradication regimens are employed in a country where the infection is highly prevalent. The data also emphasizes the necessity of performing *in vitro* susceptibility testing for rifampicin and fluoroquinolones prior to their use in the eradication of *H. pylori*.

## Figures and Tables

**Table 1:** Minimal inhibitory concentrations (MICs) of rifampin and levofloxacin on 61 *H. pylori* isolates.

MICs (mg/L)	Strains Resistant to Rifampin			Strains Resistant to Levofloxacin		
	(*n*)	(%)	95% CI	(*n*)	(%)	95% CI
≤0.124	16	26.2	16.8–38.4	39	63.9	51.4–74.8
≥0.125–0.49	10	16.4	9.15–27.6	6	9.8	4.6–19.8
≥0.50–1	27	44.3	32.5–56.7	1	1.6	0.3–8.7
>1–3.99	0	0.0	0–5.9	4	6.6	2.6–15.7
≥4–7.99	2	3.3	0.9–11.2	2	3.3	0.9–11.2
≥8–95.9	6	9.8	4.6–19.8	9	14.8	8.0–25.7
≥256	0	0.0	0–5.9	0	0.0	0–5.9
Resistance rates	8	13.1	6.8–23.8	15	24.6	15.5–36.7

95% CI—95% confidence interval for a proportion (http://www.vassarstats.net: The Confidence Interval of a Proportion). Levofloxacin and rifampin minimal inhibitory concentrations (MICs) were interpreted according to EUCAST breakpoints for resistance, >1 μg/mL.

**Table 2:** Mutations in *gyrA* gene and corresponding levofloxacin MICs.

gyrA Mutation	No. of Isolates	% of All 61 Isolates	95% CI	% of All 15 Resistant Isolates	95% CI	Levofloxacin MICs (mg/L)
N87I	1	1.6	0.3–8.7	6.7	1.2–29.8	≥32
V89A/D	1	1.6	0.3–8.7	6.7	1.2–29.8	8
Y90A/C	1	1.6	0.3–8.7	6.7	1.2–29.8	≥32
D91G/M/N	3	4.9	1.7–13.5	20.0	7.0–45.2	1.5, 3 and 4
V89A/D + Y90A/C	1	1.6	0.3–8.7	6.7	1.2–29.8	≥32
V89A/D + Y90A/C + D91G/M/N	5	8.2	3.6–17.8	33.3	15.2–58.3	4 (one isolate); ≥32 (four isolates)
No mutation detected	3	4.9	1.7–13.5	20.0	7.0–45.2	2, 3 and ≥32
Total	15	24.6	15.5–36.7	100.0	79.6–100.0	1.5–≥32

95% CI—95% confidence interval for a proportion (http://www.vassarstats.net: The Confidence Interval of a Proportion).

## Data Availability

The original contributions presented in this study are included in the article. Further inquiries can be directed to the corresponding author.
